# Is there a relationship between assisted reproductive technology and maternal outcomes? A systematic review of cohort studies

**DOI:** 10.18502/ijrm.v21i11.14651

**Published:** 2023-12-19

**Authors:** Fatemeh Heshmatnia, Maryam Jafari, Leila Bozorgian, Parvin Yadollahi, Zohre Khalajinia, Marzieh Azizi

**Affiliations:** ^1^Department of Midwifery, School of Nursing and Midwifery, Shiraz University of Medical Sciences, Shiraz, Iran.; ^2^Student Research Committee, Nursing and Midwifery School, Ahvaz Jundishapur University of Medical Sciences, Ahvaz, Iran.; ^3^Department of Midwifery, Maternal-Fetal Medicine Research Center, School of Nursing and Midwifery, Shiraz University of Medical Sciences, Shiraz, Iran.; ^4^Department of Midwifery, School of Medicine, Qom University of Medical Sciences, Qom, Iran.; ^5^Department of Midwifery, Sexual and Reproductive Health Research Center, Mazandaran University of Medical Sciences, Sari, Iran.

**Keywords:** Assisted reproductive techniques, Maternal health, Pregnancy complications, In vitro fertilization.

## Abstract

**Background:** Pregnancy with assisted reproductive technology (ART) is accompanied by fetal and maternal outcomes. **Objective:** This systematic review aimed to assess the relationship between ART and maternal outcomes. **Materials and Methods:** In this systematic review, the electronic databases, including PubMed, MEDLINE, Web of Science, Scopus, Science Direct, Cochrane Library, Google Scholar, Magiran, Irandoc, and Scientific Information Database were searched for maternal outcomes reported from 2010-2021. The Newcastle-Ottawa Scale for cohort studies was used to assess the methodological quality of studies. **Results:** A total of 3362 studies were identified by searching the databases. After screening abstracts and full-text reviews, 19 studies assessing the singleton pregnancy-related complications of in vitro fertilization/intracytoplasmic sperm injection were included in the study. The results demonstrated that singleton pregnancies conceived through ART had higher risks of pregnancy-related complications and adverse maternal outcomes, such as vaginal bleeding, cesarean section, hypertension induced by pregnancy, pre-eclampsia, placenta previa, and premature membrane rupture than those conceived naturally. **Conclusion:** In conclusion, an increased risk of adverse obstetric outcomes was observed in singleton pregnancies conceived by ART. Therefore, obstetricians should consider these pregnancies as high-risk cases and should pay special attention to their pregnancy process.

## 1. Introduction

Infertility can be defined as the failure of a couple to conceive after at least one year of having regular, unprotected intercourse (1). Nowadays, 10-20% childbearing age women are affected by infertility worldwide, and more than 15% of couples suffer from infertility worldwide (2). Similarly, several studies indicated that the prevalence of infertility in Iran has increased since 2009, with a reported rate of 20% in 2019 (3). According to the results of studies, the infertility rate in Iran is higher than the global average (4). Assisted reproductive technologies (ART) can be defined as a technique by which fertilized embryos are handled in vitro to induce pregnancy (5).

An ever-increasing population of patients seeks to conceive using ART (6). According to the studies conducted, about 1.5 million in vitro fertilization (IVF) is implemented annually. Approximately 8% of all infants born in the United States result from ART, and the total number of infants born worldwide exceeds 8 million (7-10). Moreover, 1-3% of births in Western countries and 1-5% in other countries, such as China and Japan, result from IVF (11). On the other hand, several studies indicated that the adverse consequences of ART could adversely influence the health of families and, consequently, the society (12). From the beginning of the first pregnancies resulting from ART, the health of both mothers and children conceived by ART is considered a public concern. Monitoring the data for children born after such procedures is promising, and the perinatal consequences of children conceived by ART have improved over the years; however, there is still evidence of adverse maternal and fetal consequences (6). ART-conceived women are at higher risk of pregnancy compared to those who conceived spontaneously. These consequences include ovarian hyperstimulation, preeclampsia, ectopic pregnancy, diabetes (13-16), postpartum hemorrhage (17), and twin/multiple pregnancies (18). Studies have also shown that women who have conceived using ART suffer from pregnancy-specific anxiety, lower quality of life, similar or lower symptoms of depression, and poor self-esteem (19-21). On the contrary, some studies have reported that the ART procedures associated with IVF and intracytoplasmic sperm injection (ICSI) (22) are not responsible for adverse health-related outcomes (23).

The use of ART, nowadays, is increasing, and similar to any medical intervention, it too involves a potential risk. Therefore, it is necessary to study the maternal outcomes in cases using ARTs for pregnancy. However, the literature review showed that there are contradictory studies in this regard, so this study aimed to systematically evaluate the relationship between ART and maternal outcomes.

## 2. Materials and Methods

### Design

This systematic review was based on the PRISMA guidelines (24, 25).

### Literature search and search strategy

A comprehensive literature search was conducted in electronic databases, including PubMed, MEDLINE, Web of Science, Scopus, Science Direct, Cochrane Library, Google Scholar, Magiran, Irandoc, and Scientific Information Database. The quality assessment of studies was double-checked by 2 independent researchers (F.H. and M.J.). The research process was primarily carried out based on systematic research using Persian and English keywords such as [“pregnancy” OR “pregnant women” OR “maternal outcomes” OR “pregnancy outcomes” OR “obstetric outcomes” OR “perinatal outcomes” OR “adverse maternal outcomes”] AND [“In vitro fertilization” OR “Intra cytoplasmic sperm injection” OR “assisted reproductive technology” OR “assisted reproductive technique”] AND [“cohort studies” OR “retrospective cohort” OR “prospective cohort” OR “analytical studies”]. The included studies' reference lists were manually searched to identify more relevant articles from 2010-2021. The search strategies in the mentioned databases are presented in table I.

### Inclusion and exclusion criteria

Studies eligible for inclusion in the study if they: 1) had a prospective or retrospective cohort design; 2) compared maternal outcomes of ART singleton pregnancies with those conceived naturally; 3) used IVF and/or ICSI as the exposure of interest; 4) reported maternal outcomes (or data to calculate them); and 5) were published in English and Persian languages. Review papers, non-peer-reviewed local and/or federal government reports, conference abstracts, and presentations were excluded from the study. Potential studies were evaluated to avoid the duplication of the case series.

### Data collection and extraction

The following data were extracted from the included studies: author's name, year of publication, study design (retrospective or prospective), age of participants, gestational age based on day or week, the number of participants in each group, type of ART, reports of adverse maternal outcomes and complications during pregnancy, and the main results reported by studies. 2 researchers (F.H. and M.J.) conducted independent reviews of the titles and abstracts of the included studies. The full texts of the included studies were considered for further evaluation based on the study inclusion and exclusion criteria. The 2 authors would receive consultation from the $3^{\text{rd}}$ author (M.A.) regarding any discrepancies between them.

### Methodological quality assessment

Based on the Newcastle-Ottawa Scale (NOS) principles, the quality of the studies was evaluated (26). Each quality item was awarded a star as a quick visual assessment. The number of stars determined the study quality, with 9 being given to the highest quality. Studies receiving 7 stars were considered as higher methodological quality.

NOS for cohort studies is a widely known scale for evaluating the quality and potential for bias in observational studies (27, 28). The NOS can be utilized for cross-sectional, case-control, and cohort studies (29). The NOS assesses 3 quality parameters (selection, comparability, and outcome) which are divided into 8 specific elements, differing slightly when considering cross-sectional, case-control, and cohort studies. Each scale component is graded from a point, except for the comparability parameter, which gets up to 2 points. Therefore, the maximum for each study is 9, and studies with a grade of < 5 are identified as having a high risk of bias (29, 30).

**Table 1:** The search strategies in the databases (October 2021-February 2022)


**Database**	**Latest search**	**Number of articles retrieved**	**Search filters**
**PubMed**	[“pregnancy" OR “pregnant women" OR “maternal outcome" OR “pregnancy outcome" OR “obstetric outcome" OR “perinatal outcomes" OR “adverse maternal outcomes"] AND [“In vitro fertilization" OR “Intra cytoplasmic sperm injection" OR “assisted reproductive technology" OR “assisted reproductive technique"] AND [“cohort studies" OR “retrospective cohort" OR “prospective cohort" OR “analytical studies"]	383	Species: Human Article language: English Article type: Observational study
**Scopus**	[“maternal outcomes"] AND [“In vitro fertilization" OR “Intra cytoplasmic sperm injection" OR “assisted reproductive technology" OR “assisted reproductive technique"] AND [“cohort studies" OR “retrospective cohort" OR “prospective cohort"]	385	Document types: Research articles Subject areas: Medicine Source type: Journal
**WOS**	[“maternal outcomes" OR “pregnancy outcomes" OR “obstetric outcomes"] AND [“In vitro fertilization" OR “Intra cytoplasmic sperm injection" OR “assisted reproductive technology" OR “assisted reproductive technique"] AND [“cohort studies" OR “retrospective cohort" OR “prospective cohort"]	284	Document types: Article
**Cochrane Library**	[“maternal outcomes" OR “pregnancy outcomes"] AND [“In vitro fertilization" OR “Intra cytoplasmic sperm injection" OR “assisted reproductive technology"] AND [“cohort studies" OR “retrospective cohort" OR “prospective cohort"]	180	Article language: English Persian Source: Embase, CT.gov, ICTRP, CINAHL
**Science Direct**	[“perinatal outcomes" OR “adverse maternal outcomes"] AND [“In vitro fertilization" OR “Intra cytoplasmic sperm injection" OR “assisted reproductive technology" OR “assisted reproductive technique"] AND [“cohort studies" OR “retrospective cohort" OR “prospective cohort" OR “analytical studies"]	456	Article type: Research articles Subject areas: Medicine and dentistry, nursing, and health professions Publication title: Fertility and sterility International Journal of Gynecology & Obstetrics, Midwifery, European Journal of Obstetrics & Gynecology and Reproductive Biology, Reproductive Biomedicine Online, American Journal of Obstetrics and Gynecology Access type: Open access and open archived
**Google Scholar**	[“perinatal outcomes" OR “adverse maternal outcomes"] AND [“In vitro fertilization" OR “Intra cytoplasmic sperm injection" OR “assisted reproductive technology" OR “assisted reproductive technique"] AND [“cohort studies" OR “retrospective cohort" OR “prospective cohort" OR “analytical studies"]	420	Year of publication: 2010-2022 Sort by relevance Keywords anywhere in the article
**SID**	[“Adverse maternal outcomes"] AND [“In vitro fertilization"] AND [“cohort studies" OR “retrospective cohort" OR “prospective cohort" OR “analytical studies"]	356	Subject area: Medicine Year of publication: 2010-2022
**Magiran**	[“Adverse maternal outcomes"] AND [“In vitro fertilization"] AND [“cohort studies" OR “retrospective cohort" OR “prospective cohort" OR “analytical studies"]	245	Language: Persian and English Publication type: All
**Irandoc**	[“Adverse maternal outcomes"] AND [“In vitro fertilization"] AND [“cohort studies" OR “retrospective cohort" OR “prospective cohort" OR “analytical studies"]	325	Language: Persian and English Publication type: All Year: 2010-2022
**MEDLINE**	[“Adverse maternal outcomes"] AND [“In vitro fertilization"] AND [“cohort studies" OR “retrospective cohort" OR “prospective cohort"]	328	Language: Persian and English Publication type: All Year: 2010-2022

## 3. Results

### Search results 

**Figure 1:**
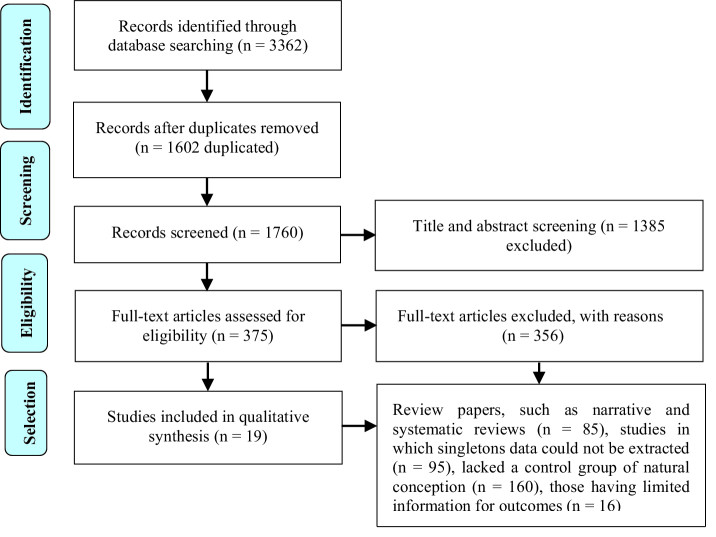
Flow chart of study selection according to PRISMA guidelines.

A total of 3362 articles were identified by using initial search criteria. 1602 out of 3362 studies were excluded due to duplication. During the title and abstract screening, 1385 records were removed. Review papers, such as narrative and systematic reviews (n = 85), studies in which singleton data could not be extracted (n = 95), lacked a control group of natural conception (n = 160), and those having limited information for outcomes (n = 16) were excluded from the study. Finally, 19 studies were included in this systematic review (Figure 1).

### Characteristics of the included studies

The characteristics of all the included studies that involved singleton births resulting from ART are summarized in table II. 8 studies were conducted in Asia (31-38), 7 in Europe (22, 39-44), 2 in the USA (45, 46), one in Australia (47), and one in Canada (48). All papers belonged to cohort studies, including 15 retrospectives (31, 33, 35-41, 43-48) and 4 prospective cohorts (22, 32, 34, 42). ART-mediated pregnancies were deﬁned as the intervention group, and control group (C.G.) pregnancies were deﬁned as the control group. The size of the exposed and unexposed cohort ranged from 23-2641 (total of 15,649) and from 88-16,335 (total of 63,965) across studies, respectively. Of the 19 included studies, 8 used the IVF method (34, 37-39, 41, 43, 46), 10 investigated IVF and ICSI method (31, 32, 33, 35, 36, 42, 44, 45, 47, 48), and one study examined ICSI (22).

### Main results 

In this systematic review, the adverse maternal outcomes of singleton pregnancy were reported by reviewing 19 studies. The main results are shown in table II.

#### Vaginal blood loss during pregnancy

This maternal outcome was assessed in 5 studies (32, 38, 39, 41, 45). All these 5 studies showed that the vaginal bleeding (V.B.) or blood loss in the IVF group was considerably higher than in CG.

The study by Koudstaal et al. showed that women with placenta previa and preterm contraction in the IVF group had higher bleeding in the second and third trimesters compared to the CG (2.3 vs. 0.3, p = 0.05) (39). According to the results of a study pregnancy complications (including spontaneous abortion, gestational diabetes mellitus (GDM), and cesarean delivery) significantly increased the risk of excess VB in ART pregnancies compared with CG (21.4% vs. 12.9%; OR = 1.67, 95% CI: 1.18-2.37) (32). The results of Schieve et al. indicated a higher risk of VB and uterine bleeding among women who had undergone the IVF compared to the CG (R.R. = 3.2, [1.5-6.8], p < 0.001) (45).

Another study reported that the first-trimester bleeding in the IVF group was higher than that in the CG (OR = 1.68; 95% CI: 1.0-2.86, p < 0.05) (41). Also, a study results, revealed that blood loss rate during delivery were 662.1 ± 6.8 mL in the CG and 998.2 ± 18.9 mL in the ART group, while the atonic bleeding in the ART group was significantly higher than that in CG (p = 0.006) (38).

#### Cesarean delivery 

Cesarean delivery as an essential maternal outcome has been reported in 12 studies (31-33, 37, 39-41, 43, 45-48). Among numbered studies, 10 studies demonstrated that the rate of cesarean delivery in the ART group was higher than that in the CG (31-33, 37, 39, 41, 43, 45-48); however, no significant differences were observed in 2 studies regarding the risk of cesarean delivery in ART group compared to CG (37, 40).

In a study which assessed the risk of prematurity in singleton pregnancy using ART, showed that a significant difference was observed concerning the prevalence rate of emergency and elective cesarean delivery between control and ART groups (28.4% vs. 14.2%, p < 0.01) and (13.7 vs. 6.3%, p = 0.04), respectively (31).

A study also demonstrated that the prevalence of elective cesarean delivery in the IVF group was significantly higher than in the CG (8.8 vs. 4.2, p = 0.03) (39). According to the results of a study the rate of cesarean delivery in the ART group was higher than that in CG (OR = 1.33, 95% CI: 0.095-1.87, p = 0.012) (41). In another 2 studies by Apantaku et al. (OR = 2.0, 95% CI: 0.7-5.8, p = 0.018) (43) and Jaques et al. (p = 0.001), the cesarean delivery rate was found to be significantly higher in ART group compared to CG (47).

The results of a study showed that the rate of cesarean delivery on maternal requests with no medical indication was significantly increased in the ART group compared to CG (OR = 1.03, 95% CI: 0.75-1.41, p < 0.05) (32). In addition, studies by Poon et al. (66.8 vs. 28.4%, OR = 4.554, 95% CI: 3.834-5.409, p < 0.05) (33), Schieve et al. (R.R. = 2.5 [2.4-2.6], p < 0.05) (45), da Silva et al. (100.0% vs. 64.7, p < 0.001) (46), and Wen et al. (38.3% vs. 30.3%, adjusted odds ratio [aOR] = 1.15, 95% CI: 0.93-1.41) (48) were other included studies, which indicated that the rate of cesarean delivery in the ART group was significantly higher than that in CG.

In a study, the results indicated no significant difference was observed between the ART and the CGs in terms of cesarean delivery (77.4% vs. 75.0%, p = 0.497) (37). Also, the study by Isaksson et al. showed no significant difference between the 2 groups for cesarean delivery (40).

#### Preterm delivery

9 studies (22, 31, 32, 37-39, 44-46) assessed preterm delivery as an adverse maternal outcome among the ART groups. In 7 studies, the prevalence of preterm delivery was higher in ART such as IVF or ICSI groups compared to CG. For example, Koudstaal et al. showed that in the IVF group, pregnancies were more likely to be terminated preterm compared to CG (15 vs. 5.9, p < 0.001) (39). Perri et al., who investigated the association between singleton ART pregnancies and the risk of prematurity indicated that preterm delivery in the ART group (20%) was significantly higher than that in CG (4%) (p = 0.001) (31). Also, Katalinic et al. revealed that preterm birth in the ICSI group was higher than that in CG (3.9% vs. 3.5%, p < 0.01) (22). Studies conducted by Schieve et al. (relative risk [RR] = 2.4, 95% CI: 1.8-3.0) (45), Farhi et al. (OR = 1.72, 95% CI: 1.04-2.87, p = 0.04) (32), Szymusik et al. (OR = 2.06; 95% CI: 1.16-3.68, p = 0.012) (44), and da Silva et al. (OR = 3.28; 95% CI: 1.32-8.13, p = 0.01) also indicated that rate of preterm labor in ART group was significantly higher than that in CG (46).

In addition, Wang et al. (37) (5.0% vs. 5.1%, p = 0.955) and Tanaka et al. (38) (aOR = 1.01, 95% CI: 0.81-1.24, p = 0.92) demonstrated that no differences were observed between the ART and CG regarding the risk of preterm labor.

#### Hypertension induced by pregnancy (HIP)

This maternal outcome was reported in 10 studies (22, 35, 37, 40-42, 44-47). Among them, 6 included studies demonstrated a higher risk of HIP in the ART group compared to the CG.

The studies by Isaksson et al., showed that the rate of HIP in CG was significantly lower than that in the IVF group (p < 0.05) (40), and da Silva et al. (46), demonstrated that a significant difference was observed between the 2 groups concerning the prevalence rate of HIP (11.1% in ART vs. 25.4% in spontaneous pregnancy, p = 0.27). Poikkeus et al. (42) reported that gestational hypertension was more typical in the ART group compared to CG (p < 0.005), and also Jaqeus et al. (47), Zhu et al. (aOR = 1.99, 95% CI: 1.56-2.53, p < 0.001) (35), Farhi et al. (32) (OR = 1.49, 95% CI: 0.93-2.38, p < 0.03), and Lei et al. (OR = 2.18, 95% CI: 1.83-2.60, p < 0.01) (36) showed that the rate of the HIP in the ART group was significantly higher than that in CG (p < 0.001).

In this regard, 4 studies showed that no statistical differences were observed in the risk of HIP among women in the ART and CGs. Katalinic et al. (RR = 1.24, 95% CI: 1.02-1.50, p = 0.47) (22) and Schieve et al. (RR = 1.5, [1.04-2.2], p < 0.11) (45) indicated that the rate of the HIP in the ART group was higher than that in CG (RR = 1.30, 95% CI: 1.11-1.52 and RR = 1.8, 95% CI: 1.4-2.2, respectively), but was not statistically significant. Other studies by Wang et al. (37) (p = 0.463), and Szymusik et al. (44) (p = 0.48) also showed no significant differences in this regard.

#### GDM

GDM as a maternal outcome was assessed in 13 studies (32, 34-37, 40-42, 44-48). In 7 studies, no increased risk of GDM was seen among the ART group compared to CG. Isaksson et al. (p > 0.05) (40), Kozinszky et al. (OR = 1.79, 95% CI: 0.83-3.81, p > 0.05) (41), Wen et al. (p > 0.05) (48), Farhi et al. (32), and da Silva et al. (46) (p = 0.20) demonstrated that no significant differences were observed between 2 groups in terms of the prevalence of GDM. In addition, the studies by Szymusik et al. (44) (p = 0.48) and Wang et al. (37) showed that GDM in the ART group was not higher than that in CG (p = 0.996).

The results of the 6 studies, including Poikkeus et al. (42) (p = 0.004), Schieve et al. (R.R. = 2.2 95% CI: 1.02-4.9, p = 0.01) (45), Jie et al. (34) (p = 0.01), Zhu et al. (35) (aOR = 2.23, 95% CI: 1.85-2.69, p < 0.001), and Lei et al. (36) (OR = 1.88, 95% CI: 1.56-2.27, p < 0.01) showed that GDM in ART group was more frequent than that in CG. Moreover, a study by Jaques et al. (47) reported weak evidence for the higher risk of GDM in the ART group compared to CG (OR = 1.25, 95% CI: 0.96-1.63, p = 0.045).

#### Pre-eclampsia or/and eclampsia 

This outcome was investigated in 10 studies (33, 35, 36, 38, 42-44, 46-48). Among the mentioned studies, 4 studies represented the higher risk of pre-eclampsia and eclampsia in ART or ICSI groups compared to CG. For example, in the studies, sub-fertile women compared to the CG experience a higher risk of preeclampsia (aOR = 1.29, 95% CI: 1.02-1.61, p < 0.01) (47). The studies by Poon et al. (OR = 2.2, 95% CI: 1.401-2.564, p < 0.05) (33) and Zhu et al. (aOR = 1.49, 95% CI: 1.12-1.98, p < 0.001) demonstrated that the women in ART group significantly experienced the pre-eclampsia compared to the CG (35).

According to the results of a study demonstrated that the risk of pre-eclampsia was increased in the ART group as compared with CG (OR = 2.15, 95% CI: 1.33-3.46), and also no significant difference rate was observed in the 2 groups in terms of eclampsia/hemolysis, elevated liver enzyme levels and low platelet levels (OR = 0.40, 95% CI: 0.14-1.09) (48).

6 other studies regarding pre-eclampsia or eclampsia as an outcome measured, showed that the risk of pre-eclampsia in the ART group was not increased compared to the C.G. For example, in a study pre-eclampsia was not increased among ART and CGs (3.5% vs. 2.2, p = 0.46) (42). In addition, Apantaku et al. (OR = 0.8, 95% CI: 0.2-2.9, p = 1.000) (43), Lei et al. (aOR = 1.57, 95% CI: 1.14-2.17, p = 0.01) (36), Szymusik et al. (OR = 1.15, 95% CI: 0.27-5.13, p = 1.0) (44), and da Silva et al. (5.6% vs. 6.4%, p = 1.00) (46) showed that no difference was observed between ART and C.G.s for the occurrence of pre-eclampsia and eclampsia (p = 0.46).

A study reported that the late onset of pre-eclampsia in the ART group was higher than that in CG, but no significant difference was observed in the early onset pre-eclampsia of the ART group (7.0% vs. 4.1%, aOR = 1.17, 95% CI: 0.83-1.63, p = 0.34) (38).

#### Placenta previa and placenta abruption 

These outcomes as 2 important maternal outcomes were assessed in 10 studies (22, 34, 36-38, 41-45). 3 studies showed that the women who had undergone ART showed no significant risk for the occurrence of placenta previa or/and abruption. For example, in the studies by Isaksson et al. (0.3% vs. 0.6%, p > 0.05), Kozinszky et al. (0.7 vs. 0.0, p > 0.05), and Wang et al. (0.7% vs. 0.4%, p > 0.999), the results showed no significant difference between the 2 groups regarding the prevalence of placenta previa or/and placenta abruption (37, 40, 41).

In a study which investigated the pregnancy course and outcome after ICSI, showed that placenta previa was higher in the ICSI group (R.R. = 5.68, 95% CI: 3.59-9.01) (44). Zhu et al. (aOR = 2.61, 95% CI: 1.78-3.83, p < 0.001) (35), and Szymusik et al. (OR = 5.15, 95% CI: 1.1-33.9, p = 0.023) (44), showed that the rate of placenta previa in ART group was higher than that in CG. In addition, based on the results of a study ART was associated with a higher rate of placenta previa (RR = 3.8, 95% CI: 1.6-9.4) and placenta abruption (RR = 3.8, 95% CI: 1.6-9.4, p < 0.001) (45).

In this regard, a study demonstrated that placenta previa and placenta abruption in C.G. were higher than those in the ART group (0.0% in C.G. vs. 0.8 in the ART group, p = 1.000) and (0.0% vs. 3.3%, p = 0.125), respectively (43).

Lei et al. showed that placenta abruption in the ART group was higher than that in CG (OR = 2.14, 95% CI: 1.33-3.45, p < 0.01). However, no significant difference was found between the 2 groups concerning the occurrence of placenta previa in singleton pregnancy (OR = 1.22, 95% CI: 0.71-2.09, p = 0.47) (36). In a study reported that placenta previa (aOR = 1.96, 95% CI: 1.24-3.06, p = 0.004) and placenta accrete (aOR = 7.35, 95% CI: 3.20-16.6, p < 0.001) in the ART group was higher than that in the CG, but the rate of placenta abruption significantly decreased in the ART group as compared with CG (aOR = 0.24, 95% CI: 0.07-0.61, p = 0.001) (38).

#### Premature rupture of membranes (PROM)

This maternal outcome including polyhydramnios and oligohydramnios were assessed in 9 studies (22, 33, 35, 36, 41-44, 47).

5 studies showed no differences between the 2 groups in this regard. For example, the results of a study showed that PROM in the ART group was not higher than that in CG (OR = 3.73, 95% CI: 0.73-25.63, p = 0.11) (44). Also, the study showed that no significant difference was observed between the 2 groups in terms of polyhydramnios (OR = 0.43, 95% CI: 0.19-0.97) and oligohydramnios (OR = 1.58, 95% CI: 0.77-3.24) rates (41). Another study showed that PROM (aOR = 3.05, 95% CI: 2.48-3.74, p < 0.001) and polyhydramnios (aOR = 1.79, 95% CI: 1.26-2.53, p = 0.03) were more prevalent in the ART group, but no significant difference was observed between the 2 groups regarding the oligohydramnios rates (aOR = 0.67, 95% CI: 0.54-0.83, p = 0.572) (35). A study assessed the amniotic fluid embolism (aOR = 5.93, 95% CI: 0.59-55.43, p = 0.12) and uterine rupture (aOR = 3.09, 95% CI: 0.13-30.16, p = 0.40) between the 2 groups and found that no significant differences were observed in this regard (38). Consistent with the above mentioned studies, a study demonstrated that no significant difference was observed between the 2 groups in terms of the PROPM rate (0.7% vs. 0.35, p = 0.25) and (5.8% vs. 5.0% p = 0.5) respectively (43). 2 studies found no differences in these 3 factors in the 2 groups (p < 0.05) (33, 47).

In a, the prevalence rates of PROM (p = 0.03), polyhydramnios (p = 0.01), and oligohydramnios (p = 0.20) in the ART group were found to be higher than those in the CG (36). The results of the study by Katalinic et al. demonstrated that PROM in the ART group was lower than that in CG (RR = 0.88, 95% CI: 0.78-1.00), but polyhydramnios (RR = 1.56, 95% CI: 0.95-2.55) and oligohydramnios (RR = 2.14, 95% CI: 1.51-3.03) rates in ART group were higher than those in CG (22).

### Quality assessment of the included studies

The quality of 19 studies were assessed using the NOS for cohort studies. The results showed that all the included studies had good methodological quality. The quality assessment of the included studies is presented in table III.

**Table 2:** The characteristics of the included studies


**Author, Year (Ref)**	**Study design**	**Age (mean ± S.D.)**	**Gestational Age/week or day**	**Sample size in each group**	**Type of ART**	**Pregnancy-related complications and adverse outcomes: n/N (%)**	**Main result**
**Koudstaal ** ***et al.*** **, 2000 (39)**	Retrospective cohort	I.G.: 32.80 ± 4.30 CG: 32.70 ± 4.40	> 16	I.G.: 307 CG: 307	IVF	IG, CG EmC: 7.80, EmC: 8.8 EC: 8.8, EC: 4.2 NVD: 67.4, NVD: 63.2 IVD: 16, IVD: 23.8 FT: 21.2, FT: 13.7 ST: 7.8, ST: 2 TT: 8.6, TT: 0.03 PD: 15, PD: 5.9	The C-section (8.8 vs. 4.2, p = 0.03) and preterm delivery (15 vs. 5.9, p < 0.001) bleeding in the second and third trimesters (2.3 vs. 0.3, p = 0.05) were significantly higher in the IVF groups compared to the CG
**Perri ** ***et al.*** **, 2001 (31)**	Retrospective cohort	I.G.: 32.15 ± 4.50 CG: 32.12 ± 4.50	> 23	I.G.: 95 C.G.: 190	IVF ICSI	IG, CG EmC: 27 (28.4), EmC: 27 (14.2) EC: 13 (13.7), EC: 12 (6.3) SVD: 53 (55.8), SVD: 139 (73.2) IVD: 2 (2.1), IVD: 12 (6.3)	Pregnancies under ART had an increased risk of giving birth to premature delivery (p = 0.001) and emergency CS (p < 0.01) compared to pregnancies following spontaneous conception
**Isaksson ** ***et al.*** **, 2000 (40)**	Retrospective cohort	I.G.: 33.80 ± 3.20 CG: 33.80 ± 3.20	IG1: 39.70 ± 2.10 IG2: 39.40 ± 2.10 C.G.: 39.30 ± 2.10	IG: 69 CG1: 345 CG2: 1901	IVF	IG, CG2 PP: 0, PP: 0.6 PA: 1.4, PA: 0.3 HIP: 0, HIP: 6.4 GDM: 0, GDM: 1.4 CS: 24.6, CS: 28.7	The overall outcome of obstetrics such as GDM, CS, and PP in couples with unexplained infertility treated with IVF had similar results in comparison to spontaneous pregnancies (p > 0.05). The rate of HIP in CG was significantly lower than that in the IVF group (p < 0.05)
**Kozinszky ** ***et al.*** **, 2003 (41)**	Retrospective cohort	I.G.: 32.30 ± 4.00 CG: 32.00 ± 4.10	N/A	IG: 284 CG: 284	IVF	IG, CG GDM: 19 (6.7), GDM: 11 (3.9) PP: 2 (0.7), PP: 0 HIP: 0, HIP: 0 IUI: 18 (6.3), IUI: 3 (10.6) Oligohydramnios: 9 (3.2) Oligohydramnios: 20 (7) Polyhydramnios: 2 (0.7) Polyhydramnios: 2 (0.7) PROM: 92 (32.4), PROM: 114 (40.1) CS: 98 (34.5), CS: 117 (41.2)	The rate of C-sections in the ART group was higher than that in CG (OR = 1.33, 95% CI: 0.095-1.87, p = 0.012), PROM (OR = 0.43, 95% CI: 0.19-0.97) and oligohydramnios (OR = 1.58, 95% CI: 0.77-3.24) rates
**Katalinic ** ***et al.*** **, 2004 (22)**	Prospective cohort	IG: 32.90 ± 3.90 CG: 27.00 ± 4.70	> 28	I.G.: 2055 CG: 7861	ICSI	IG, CG VB before $28^{\text{th}}$ wk: 511 ± 24.90, VB before $28^{\text{th}}$ wk: 514 (6.5) VB after $28^{\text{th}}$ wk: 80 (3.9), VB after $28^{\text{th}}$ wk: 80 (1.0) PP: 47 (2.3), PP: 28 (0.4) Polyhydramnios: 22 (1.1) Polyhydramnios: 54 (0.7) Oligohydramnios: 48 (2.3) Oligohydramnios: 86 (1.1) PTL: 434 (21.1), PTL: 640 (8.1) PA: 42 (2.0), PA: 89 (1.1) PROM: 262 (12.7), PROM: 1135 (14.4) Anemia: 621 (30.2), Anemia: 1417 (18.0) HT: 193 (9.4), HT: 569 (7.2) Premature birth: 248 (12.1) Premature birth: 524 (6.7)	Placenta previa (R.R. = 5.68, 95% CI: 3.59, 9.01) and preterm birth (3.9% vs. 3.5%, p < 0.01) were higher in the ICSI group
**Poikkeus ** ***et al.*** **, 2007 (42)**	Prospective cohort	I.G.: 23-42 C.G.: 14-51	N/A	IG: 499 CG: 15037	IVF	IG, CG GH: 28 (0.8), GH: 349 (2.3) PE: 13 (10.7), PE: 246 (1.6) GDM: 46 (17.8), GDM: 924 (6.1) IHC: 8 (2.11), IHC: 151 (1.0) PP: 13 (4.12), PP: 65 (0.4) PA: 5 (2.2), PA: 59 (0.4) PROM: 52 (0.35), PROM: 6 (1.14)	Compared to the control group, GDM (p = 0.004) and GH (p < 0.005) were more frequent in the ART group
**Schieve ** ***et al.*** **, 2007 (45)**	Retrospective cohort	I.G.: 42-57 CG: 42-57	N/A	IG: 1400 CG: 1400	IVF ICSI	IG, CG GDM: 4.23, GDM: 2.93 HIP: 4.95, HIP: 3.29 VB: 2.08, VB: 0.64 PA: 1.65, PA: 0.43 PP: 1.65, PP: 0.43 CS: 31.61, CS: 28.17 PTD: 13.89, PTD: 5.87	A higher rate of PP (R.R. = 3.8, 95% CI: 1.6-9.4), GDM (R.R. = 2.2, 95% CI: 1.02-4.9), HIP (R.R. = 1.5, [1.04-2.2], p < 0.01), PTD (RR = 2.4, 95% CI: 1.8-3.0) and VB (RR = 3.2, [1.5-6.8], p < 0.001) in ART group compared to CG was observed
**Apantaku ** ***et al.*** **, 2008 (43)**	Retrospective cohort	IG: 33.50 ± 4.00 CG: 33.20 ± 4.10	IG: 40 ± 10 CG: 39 ± 30	I.G.: 88 CG: 88	IVF	IG, CG PROM: 7 (5.8), PROM: 6 (5.0) PE: 6 (6.8), PE: 7 (7.9) PA: 0, PA: 4 (3.3) PP: 0, PP: 1 (0.8) CS: 18 (20.4%), CS: 11 (12.4)	Women had no excessive obstetric complications compared to the control group. However, they tended to have higher C-section levels (OR = 2.0, 95% CI: 0.7-5.8, p = 0.018)
**Wen ** ***et al.*** **, 2010 (48)**	Retrospective cohort	IG: 35.10 ± 3.70 CG: 33.80 ± 4.00	> 20	I.G.: 809 CG: 1505	IVF ICSI	IG, CG PE: 63 (7.81), PE: 37 (2.46) Eclampsia/HELLP: 6 (0.74) Eclampsia/HELLP: 18 (1.20) GDM: 41 (5.10), GDM: 81 (5.38) CS: 310 (38.32), CS: 456 (30.30)	The risk of CS (38.3% vs. 30.3%, aOR = 1.15, 95% CI: 0.93-1.41) and pre-eclampsia (OR = 2.15, 95% CI: 1.33-3.46) was increased in the ART group as compared with CG
**Jaques ** ***et al.*** **, 2010 (47)**	Retrospective cohort	IG: 33.20 ± 4.60 CG: 33.20 ± 4.60	N/A	IG: 2171 CG: 4363	IVF ICSI	IG, CG HIP/PE: 178 (8.2), HIP/PE: 207 (4.7) AH: 98 (4.5), AH: 150 (3.4) PROM: 176 (8.1), PROM: 252 (5.8) GDM: 113 (5.2), GDM: 192 (4.4) CS: 764 (35.2), CS: 995 (22.8)	The C-section rate was significantly higher in the ART group compared to CG (p = 0.001)
**Farhi ** ***et al.*** **, 2013 (32)**	Prospective cohort	I.G.: 17-41 CG: 17-41	N/A	IG: 561 CG: 600	IVF ICSI	IG, CG VB: 109.509 (21.4), VB: 76/587 (12.9) HIP: 39.587 (6.6), HIP: 39/587 (6.6) GDM: 61.509 (12.0), GDM: 59/587 (10.1) CS: 133/509 (26.1), CS: 125/587 (21.3) PTD: 54/509 (10.6), PTD: 31/587 (5.3)	The risk of HIP (OR = 1.49, 95% CI: 0.93-2.38, p < 0.03), PTD (OR = 1.72, 95% CI: 1.04-2.87, p = 0.04), CS (OR = 1.03, 95% CI: 0.75-1.41, p < 0.05) and VB (21.4% vs.12.9%; OR = 1.67, 95% CI: 1.18-2.37) in ART group was higher than CG
**Szymusik ** ***et al.*** **, 2019 (44)**	Retrospective cohort	IG: 33.90 ± 3.80 CG: 33.60 ± 3.80	IG: 38.10 ± 2.30 CG: 38.90 ± 1.60	IG: 336 CG: 308	IVF ICSI	IG, CG F.T. bleeding: 47 (13.99), F.T. bleeding: 27 (8.8) GDM: 41 (12.2), GDM: 44 (14.37) HIP: 26 (7.73), HIP: 20 (6.5) PE: 5 (1.5), PE: 4 (1.3) P.P.: 11 (3.2), P.P.: 2 (0.65) IHC: 6 (1.78), IHC: 3 (0.98) PROM: 8 (2.38), PROM: 2 (0.65) PTD: 44 (13.24), PTD: 21 (6.81)	Compared to the CG, PP (OR = 5.15, 95% CI: 1.1-33.9, p = 0.023) and PTD (OR = 2.06; 95% CI: 1.16-3.68, p = 0.012) were higher in the ART group
**Wang, ** ***et al.*** **, 2021 (37)**	Retrospective cohort	IG: 33.36 ± 5.20 CG: 33.32 ± 5.16	N/A	IG: 535 CG: 1605	IVF	IG, CG CS: 154/199 (77.4), CS: 453/604 (75.0) PP: 1/271 (0.7), PP: 3/766 (0.4) HIP: 9/271 (3.3), HIP: 19/766 (2.5) GDM: 6/271 (2.2), GDM: 17/766 (2.2) PROM: 3/271 (1.1), PROM: 4/766 (0.5) PTD: 27/535 (5.0), PTD: 82/1605 (5.1) EP: 5/271 (1.8), EP: 21/766 (2.7) CM: 72/271 (26.6), CM: 159/766 (20.8)	There was no significant difference between the groups in terms of pregnancy indicators (p > 0.05)
**Tanaka ** ***et al.*** **, 2020 (38)**	Retrospective cohort	IG: 37.20 ± 0.10 CG: 31.00 ± 0.20	IG: 37.40 ± 0.10 CG: 37.50 ± 0.10	IG: 556 CG: 2956	IVF	IG, CG PTD: 178 (22.5), PTD: 1274 (21.5) Early onset PE: 13 (1.6), Early onset PE: 79 (1.3) Late onset PE: 57 (7.0), Late onset PE: 252 (4.1) LLP: 21 (2.6), LLP: 79 (1.3) PP: 34 (4.2), PP: 96 (1.6) Placenta accrete: 18 (2.6) Placenta accrete: 18 (0.3) PA: 4 (0.5), PA: 103 (1.7) Atonic bleeding: 31 (3.8) Atonic bleeding: 103 (1.7) UR: 1 (1.0), UR: 4 (0.006) AFE: 2 (0.2), AFE: 3 (0.005)	P.P. (aOR = 1.96, 95% CI: 1.24-3.06, p = 0.004) and VB (p = 0.006) in the ART group were higher than that in the CG, but the rate of PA significantly decreased in the ART group as compared with the CG (aOR = 0.24, 95% CI: 0.07-0.61, p = 0.001)
**da Silva ** ***et al.*** **, 2020 (46)(47)**	Retrospective cohort	IG: < 30: 2 30-36: 8 36-39: 5 > 40: 2 CG: < 30: 2613 30-35: 1130 36-39: 350 > 40: 125	IG: 36.90 ± 2.00 CG: 38.50 ± 2.30	IG: 23 C.G.: 4252	IVF	IG, CG CS: 18 (100.0), CS: 2717 (64.7) HIP: 2 (11.1), HIP: 1066 (25.4) PE: 1 (5.6), PE: 266 (6.4) GDM: 3 (16.7), GDM: 358 (8.5) Hospitalization: 4 (22.2) Hospitalization: 829 (19.7) PTD: 34.8, PTD: 15.4	PTL (OR = 3.28; 95% CI: 1.32-8.13, p = 0.01) and C.S. (100.0% vs. 64.7, p < 0.001) was significantly higher than that in CG
ART: Assisted reproductive technique, IG: Intervention group, CG: Control group, EmC: Emergency cesarean, EC: Elective cesarean, CS: Cesarean section, GDM: Gestational diabetes mellitus, HIP: Hypertension induced by pregnancy, PE: Pre-eclampsia, IVD: Instrumental vaginal delivery, NVD: Normal vaginal delivery, FT: First trimester, ST: Second trimester, TT: Third trimester, PTD: Preterm delivery, VB: Vaginal bleeding, PP: Placenta previa, PA: Placenta abruption, IUI: Intra uterine infection, PROM: Preterm rapture of membrane, PTL: Preterm labor, GH: Gestational hypertension, LLP: Low lying placenta, UR: Uterine rapture, AFE: Amniotic fluid embolismic, IHC: Intrahepatic cholestasis, EP: Ectopic pregnancy, CM: Clinical miscarriage, AH: Antepartum hemorrhage, IVF: In vitro fertilization, ICSI: Intra cytoplasmic sperm injection, HELLP: Hemolysis, elevated liver enzyme levels and low platelet levels, aOR: Adjusted odds ratio, PD: Peritoneal dialysis, SVD: Spontaneous vaginal delivery, N/A: Not applicable							

**Table 3:** The methodological quality assessment of the included studies


**Author, year (Ref)**	**Representativeness of samples**	**Sample size**	**Non-respondent**	**Ascertainment of the exposure**	**Compatibility of the subjects in different outcome groups were compared based on the study design or analysis. Confounding factors were controlled**	**Assessment of the outcomes**	**Statistical test**	**Score (0-10 stars)**
**Koudstall ** ***et al.*** **, 2000 (39)**	A*	A*	A*	A**	A*	C*	A*	8
**Perri ** ***et al.*** **, 2001 (31)**	B*	B*	A*	A**	A*	C*	A*	8
**Isaksson ** ***et al.*** **, 2000 (40)**	A*	A*	A*	A**	A*	C*	A*	8
**Kozinszky ** ***et al.*** **, 2003 (41)**	A*	a*	A*	A**	A*	C*	a*	8
**Katalinic ** ***et al.*** **, 2004 (22)**	A*	A*	A*	A**	A*	C*	A*	8
**Poikkeus ** ***et al.*** **, 2007 (42)**	A*	A*	A*	A**	A*	C*	A*	8
**Schieve ** ***et al.*** **, 2007 (45)**	a*	a*	A*	A**	a*	C*	A*	8
**Apantaku ** ***et al.*** **, 2008 (43)**	B*	B*	A*	B*	A*	C*	A*	7
**Wen ** ***et al.*** **, 2010 (48)**	A*	A*	A*	A**	A*	C*	A*	8
**Jaques ** ***et al.*** **, 2010 (47)**	B*	A*	c	A**	A*	C*	A*	7
**Farhi ** ***et al.*** **, 2013 (32)**	B*	A*	A*	A**	A*	C*	A*	8
**Poon ** ***et al.*** **, 2013 (33)**	B*	A*	A*	A**	A*	C*	A*	8
**Jie ** ***et al.*** **, 2015 (34)**	B*	A*	A*	A**	A*	C*	A*	8
**Zhu ** ***et al.*** **, 2016 (35)**	A*	A*	A*	A**	A*	C*	A*	8
**Lei ** ***et al.*** **, 2019 (36)**	A*	A*	B	A*	A**	C*	A*	7
**Szymusik ** ***et al.*** **, 2019 (44)**	B*	A*	B	A*	A**	C*	A*	7
**Wang ** ***et al.*** **, 2021 (37)**	B*	A*	A*	A**	A*	C*	A*	8
**Tanaka ** ***et al.*** **, 2020 (38)**	A*	A*	A*	A**	A*	C*	A*	8
**da Silva ** ***et al.*** **, 2020 (46)**	A*	A*	A*	A**	A*	C*	A*	8
NEWCASTLE–OTTAWA QUALITY ASSESSMENT SCALE. Selection: (Maximum 5 stars). 1) Representativeness of the sample a) Truly representative of the average in the target population.* (all subjects or random sampling) b) Somewhat representative of the average in the target population. *(non-random sampling) 2) Sample size: a) Justified and satisfactory.* b) Not justified 3) Non-respondents: a) Comparability between respondents and non-respondents characteristics is established, and the response rate is satisfactory.* b) The response rate is unsatisfactory, or the comparability between respondents and non-respondents is unsatisfactory 4) Ascertainment of the exposure (Addis et al.): a) Validated measurement tool.** b) Non-validated measurement tool, but the tool is available or described.* Comparability: (Maximum 2 stars) 1) The subjects in different outcome groups are comparable, based on the study design or analysis. Confounding factors are controlled a) The study controls for the most important factor (select one).* b) The study control for any additional factor.* Outcome: (Maximum 3 stars) 1) Assessment of the outcome: a) Independent blind assessment.** b) Record linkage.** c) Self report.* 2) Statistical test: a) The statistical test used to analyze the data is clearly described and appropriate, and the measurement of the association is presented, including confidence intervals and the probability level (p value).* b) The statistical test is not appropriate, not described, or incomplete								

## 4. Discussion

This study aimed to assess whether several studies agree with the maternal complication of ART. Literature review showed that adverse maternal outcomes in IVF singleton pregnancies, including VB (32, 38, 41, 45), cesarean section (32, 33, 41, 43, 45-48), hypertension (32, 35, 36, 42, 44, 47), GDM (34-36, 42, 45), pre-eclampsia (33-35, 38, 47, 48), placenta previa (22, 34, 35, 42, 43, 45), PROM (22, 34-36, 43, 44) are increased. In contrast to our study, some studies showed opposite results. For example, Poikkeus et al. and Jaqeus et al. showed that no significant differences were observed between the 2 groups in terms of PROM (42, 47). Also, a study showed that no significant difference was found between ART and control groups in terms of pre-eclampsia and eclampsia (42). A study reported no significant difference between the 2 groups regarding preterm delivery (38). Isaksson et al. found that no significant difference was observed between the 2 groups in terms of cesarean delivery rate (40). Results of 3 studies indicated weak evidence for GDM in 2 groups (37, 44, 47). According to the results of a study no significant difference was observed between the 2 groups in terms of the occurrence of placenta previa in singleton pregnancy (36).

It is well known that ART pregnancies have an increased risk of maternal in comparison with naturally conceived pregnancies, whether singletons or multiples (49). In line with the present study, some studies have shown that most ART pregnancies are associated with higher maternal risks (50-52). However, recent advancement in ART caused some controversies, so that the singletons may be associated with higher obstetric risks due to how they are done. Researchers are becoming increasingly interested in this topic as single-embryo transfers become more common (53).

It is unclear why ART singleton pregnancies lead to an increase in adverse pregnancy outcomes (APOs) prevalence; however, some studies suggest that ART procedures, maternal infertility factors, or a combination of these factors can contribute to infertility (54). Researchers found that factors associated with ART itself, such as induce ovulation medications or maintain pregnancy early in the pregnancy cycle, the duration of culture, freezing and thawing embryos, polyspermic fertilization, and delayed oocyte fertilization, may change the hormonal environment at the time of implantation, failing to implant. Both gametes and embryos can be manipulated to produce APOs. Also, the higher rates of APOs observed in ART pregnancies may be attributable to closer monitoring of pregnancies made through ART than those conceived naturally (47, 54-56). In contrast, there are fewer studies suggesting that ART procedures, such as IVF and ICSI, are not responsible for APOs (40). These complications can be seen in sub-fertile women who conceived without the aid of ART, but they experienced a higher risk of preterm birth (57-61), pregnancy-induced hypertension or pre-eclampsia (60-62), and GDM (60). In addition to the above reason, parents who use ART have higher socioeconomic status (63). Couples who have undergone IVF and/or ICSI, especially those with severe conditions requiring more invasive procedures and common monitoring sessions, have a stronger desire for experiencing healthy pregnancy (48). So, there are more reports of their complaints. Finally, there are other reasons for differences in outcomes prevalence among continents and countries, such as ethnic, socioeconomic, and environmental differences, medical insurance, screening programs, sample size, medical procedures, study design, data collection methods, etc., which may contribute to these differences.

### Clinical implication

This study can be a useful resource for midwives and gynecologists to understand the most common maternal outcomes among couples using ART for pregnancy and pay considerable attention to these women during pregnancy. Also, the findings of this systematic review showed the importance of conducting high-quality studies to improve the physical health of pregnant women.

### Strength and limitations

This study had 2 limitations, including a lack of evaluation of the prevalence of maternal complications based on the type of pregnancy (singleton vs. twin pregnancy) and using only English and Persian full-text papers. The strengths of this study were focused solely on IVF and/or ICSI outcomes, leading to better complication assessment and management.

## 5. Conclusion

This study aimed to determine whether singleton pregnancies resulting from ART are more likely than spontaneous conceptions to lead to adverse maternal outcomes. The results of the present study demonstrated that singleton pregnancies conceived through ART are more likely to experience complications and adverse pregnancy outcomes than those conceived naturally. In addition to the clinical implications, a better understanding of this issue may provide useful information for counseling ART patients. Further research is needed to determine which aspects of ART pose the most significant risk and how to minimize this risk.

##  Conflict of Interest

The authors declare that there is no conflict of interest.

## References

[B1] Babakhanzadeh E, Nazari M, Ghasemifar S, Khodadadian A (2020). Some of the factors involved in male infertility: A prospective review. Int J Gen Med.

[B2] Hu K-L, Ye X, Wang S, Zhang D (2020). Melatonin application in assisted reproductive technology: A systematic review and meta-analysis of randomized trials. Front Endocrinol.

[B3] Maharlouei N, Morshed Behbahani B, Doryanizadeh L, Kazemi M (2021). Prevalence and pattern of infertility in Iran: A systematic review and meta-analysis study. Women Health Bull.

[B4] Akhondi MM, Ranjbar F, Shirzad M, Behjati Ardakani Z, Kamali K, Mohammad K (2019). Practical difficulties in estimating the prevalence of primary infertility in Iran. Int J Fertil Steril.

[B5] Li F, Niu AQ, Feng XM, Yan Y, Chen Y (2021). The threshold effect of factors associated with spontaneous abortion in human-assisted reproductive technology. Sci Rep.

[B6] Rodriguez-Wallberg KA, Lundberg FE, Ekberg S, Johansson AL, Ludvigsson JF, Almqvist C, et al (2020). Mortality from infancy to adolescence in singleton children conceived from assisted reproductive techniques versus naturally conceived singletons in Sweden. Fertil Steril.

[B7] Zhang L, Zhang W, Xu H, Liu K (2021). Birth defects surveillance after assisted reproductive technology in Beijing: A whole of population-based cohort study. BMJ Open.

[B8] Chang J, Zhang Y, Boulet ShL, Crawford SB, Copeland GE, Bernson D, et al (2023). Assisted reproductive technology and perinatal mortality: Selected states (2006-2011). Am J Perinatol.

[B9] Norrman E (2020). Long-term outcome of children born after assisted reproductive technology [thesis].

[B10] Wennerholm U-B, Bergh Ch (2020). Perinatal outcome in children born after assisted reproductive technologies. Ups J Med Sci.

[B11] Cui L, Zhou W, Xi B, Ma J, Hu J, Fang M, et al (2020). Increased risk of metabolic dysfunction in children conceived by assisted reproductive technology. Diabetologia.

[B12] Han Y, Luo H, Zhang Y (2018). Congenital anomalies in infants conceived by infertile women through assisted reproductive technology: A cohort study 2004‑2014. Exp Ther Med.

[B13] Opdahl S, Henningsen AA, Tiitinen A, Bergh C, Pinborg A, Romundstad P, et al (2015). Risk of hypertensive disorders in pregnancies following assisted reproductive technology: A cohort study from the CoNARTaS group. Hum Reprod.

[B14] Morency A-M, Shah PS, Seaward PGR, Whittle W, Murphy KE (2016). Obstetrical and neonatal outcomes of triplet births-spontaneous versus assisted reproductive technology conception. J Matern Fetal Neonatal Med.

[B15] Jin X-Y, Li Ch, Xu W, Liu L, Wei M-L, Fei H-Y, et al (2020). Factors associated with the incidence of ectopic pregnancy in women undergoing assisted reproductive treatment. Chin Med J.

[B16] Berntsen S, Pinborg A (2018). Large for gestational age and macrosomia in singletons born after frozen/thawed embryo transfer (FET) in assisted reproductive technology (ART). Birth Defects Res.

[B17] Cromi A, Candeloro I, Marconi N, Casarin J, Serati M, Agosti M, et al (2016). Risk of peripartum hysterectomy in births after assisted reproductive technology. Fertil Steril.

[B18] Rashid D, Alalaf S (2020). Maternal and perinatal outcomes in twin pregnancies conceived spontaneously and by assisted reproductive techniques: Cross-sectional study. East Mediterr Health J.

[B19] Gourounti K (2016). Psychological stress and adjustment in pregnancy following assisted reproductive technology and spontaneous conception: A systematic review. Women Health.

[B20] Gdańska P, Drozdowicz-Jastrzębska E, Grzechocińska B, Radziwon-Zaleska M, Węgrzyn P, Wielgoś M (2017). Anxiety and depression in women undergoing infertility treatment. Ginekol Pol.

[B21] Nicoloro-SantaBarbara J, Busso C, Moyer A, Lobel M (2018). Just relax and you'll get pregnant? Meta-analysis examining women's emotional distress and the outcome of assisted reproductive technology. Soc Sci Med.

[B22] Katalinic A, Rösch C, Ludwig M, Group GIF-US (2004). Pregnancy course and outcome after intracytoplasmic sperm injection: A controlled, prospective cohort study. Fertil Steril.

[B23] Mozafari Kermani R, Farhangniya M, Shahzadeh Fazeli SA, Bagheri P, Ashrafi M, Vosough Taqi Dizaj A (2018). Congenital malformations in singleton infants conceived by assisted reproductive technologies and singleton infants by natural conception in Tehran, Iran. Int J Fertil Steril.

[B24] Liberati A, Altman DG, Tetzlaff J, Mulrow C, Gøtzsche PC, Ioannidis JP, et al (2009). The PRISMA statement for reporting systematic reviews and meta-analyses of studies that evaluate health care interventions: Explanation and elaboration. PLoS Med.

[B25] Moher D, Shamseer L, Clarke M, Ghersi D, Liberati A, Petticrew M, et al (2015). Preferred reporting items for systematic review and meta-analysis protocols (PRISMA-P) 2015 statement. Syst Rev.

[B26] Wells GA, Shea B, O’Connell D, Peterson J, Welch V, Losos M, et al https://api.semanticscholar.org/CorpusID:79550924.

[B27] Stang A (2010). Critical evaluation of the Newcastle-Ottawa Scale for the assessment of the quality of nonrandomized studies in meta-analyses. Eur J Epidemiol.

[B28] Margulis AV, Pladevall M, Riera-Guardia N, Varas-Lorenzo C, Hazell L, Berkman ND, et al (2014). Quality assessment of observational studies in a drug-safety systematic review, comparison of two tools: The Newcastle-Ottawa scale and the RTI item bank. Clin Epidemiol.

[B29] Peterson J, Welch V, Losos M, Tugwell PJ (2011). The Newcastle-Ottawa scale (NOS) for assessing the quality of nonrandomised studies in meta-analyses.

[B30] Luchini C, Stubbs B, Solmi M, Veronese N (2017). Assessing the quality of studies in meta-analyses: Advantages and limitations of the Newcastle Ottawa Scale. World J Meta-Anal.

[B31] Perri T, Chen R, Yoeli R, Merlob P, Orvieto R, Shalev Y, et al

[B32] Farhi A, Reichman B, Boyko V, Hourvitz A, Ron-El R, Lerner-Geva L (2013). Maternal and neonatal health outcomes following assisted reproduction. Reprod Biomed Online.

[B33] Poon WB, Lian WB (2013). Perinatal outcomes of intrauterine insemination/clomiphene pregnancies represent an intermediate risk group compared with in vitro fertilisation/intracytoplasmic sperm injection and naturally conceived pregnancies. J Paediatr Child Health.

[B34] Jie Zh, Yiling D, Ling Y (2015). Association of assisted reproductive technology with adverse pregnancy outcomes. Iran J Reprod Med.

[B35] Zhu L, Zhang Y, Liu Y, Zhang R, Wu Y, Huang Y, et al (2016). Maternal and live-birth outcomes of pregnancies following assisted reproductive technology: A retrospective cohort study. Sci Rep.

[B36] Lei L-L, Lan Y-L, Wang S-Y, Feng W, Zhai Z-J (2019). Perinatal complications and live-birth outcomes following assisted reproductive technology: A retrospective cohort study. Chin Med J.

[B37] Wang Y, Yao Z, Zhao H, Yue C, Yu Q, Zhang Y, et al (2021). Reproductive outcomes of in vitro fertilization-intracytoplasmic sperm injection after transcervical resection of adhesions: A retrospective cohort study. J Minim Invasive Gynecol.

[B38] Tanaka H, Tanaka K, Osato K, Kusaka H, Maegawa Y, Taniguchi H, et al (2020). Evaluation of maternal and neonatal outcomes of assisted reproduction technology: A retrospective cohort study. Medicina.

[B39] Koudstaal J, Braat D, Bruinse H, Naaktgeboren N, Vermeiden J, Visser G (2000). Obstetric outcome of singleton pregnancies after IVF: A matched control study in four Dutch university hospitals. Hum Reprod.

[B40] Isaksson R, Gissler M, Tiitinen A (2000). Obstetric outcome among women with unexplained infertility after IVF: A matched case-control study. Hum Reprod.

[B41] Kozinszky Z, Zádori J, Orvos H, Katona M, Pál A, Kovács L (2003). Obstetric and neonatal risk of pregnancies after assisted reproductive technology: A matched control study. Acta Obstet Gynecol Scand.

[B42] Poikkeus P, Gissler M, Unkila-Kallio L, Hyden-Granskog C, Tiitinen A (2007). Obstetric and neonatal outcome after single embryo transfer. Hum Reprod.

[B43] Apantaku O, Chandrasekaran I, Bentick B (2008). Obstetric outcome of singleton pregnancies achieved with in vitro fertilisation and intracytoplasmic sperm injection: Experience from a district general hospital. J Obstet Gynaecol.

[B44] Szymusik I, Kosinska-Kaczynska K, Krowicka M, Sep M, Marianowski P, Wielgos M (2019). Perinatal outcome of in vitro fertilization singletons-10 years' experience of one center. Arch Med Sci.

[B45] Schieve LA, Cohen B, Nannini A, Ferre C, Reynolds MA, Zhang Z, et al (2007). A population-based study of maternal and perinatal outcomes associated with assisted reproductive technology in Massachusetts. Matern Child Health J.

[B46] da Silva SG, da Silveira MF, Bertoldi AD, Domingues MR, Dos Santos IDS (2020). Maternal and child-health outcomes in pregnancies following assisted reproductive technology (ART): A prospective cohort study. BMC Pregnancy Childbirth.

[B47] Jaques AM, Amor DJ, Baker HG, Healy DL, Ukoumunne OC, Breheny S, et al (2010). Adverse obstetric and perinatal outcomes in subfertile women conceiving without assisted reproductive technologies. Fertil Steril.

[B48] Wen ShW, Leader A, White RR, Léveillé M-C, Wilkie V, Zhou J, et al (2010). A comprehensive assessment of outcomes in pregnancies conceived by in vitro fertilization/intracytoplasmic sperm injection. Eur J Obstet Gynecol Reprod Biol.

[B49] Qin J-B, Sheng X-Q, Wu D, Gao Sh-Y, You Y-P, Yang T-B, et al (2017). Worldwide prevalence of adverse pregnancy outcomes among singleton pregnancies after in vitro fertilization/intracytoplasmic sperm injection: A systematic review and meta-analysis. Arch Gynecol Obstet.

[B50] Chih HJ, Elias FTS, Gaudet L, Velez MP (2021). Assisted reproductive technology and hypertensive disorders of pregnancy: Systematic review and meta-analyses. BMC Pregnancy Childbirth.

[B51] Nyfløt LT, Sandven I, Oldereid NB, Stray-Pedersen B, Vangen S (2017). Assisted reproductive technology and severe postpartum haemorrhage: A case-control study. BJOG.

[B52] Luke B, Brown MB, Wantman E, Forestieri NE, Browne ML, Fisher SC, et al (2021). The risk of birth defects with conception by ART. Hum Reprod.

[B53] Bosch E, De Vos M, Humaidan P (2020). The future of cryopreservation in assisted reproductive technologies. Front Endocrinol.

[B54] Hansen M, Kurinczuk JJ, Bower C, Webb S (2002). The risk of major birth defects after intracytoplasmic sperm injection and in vitro fertilization. N Engl J Med.

[B55] Hansen M, Kurinczuk JJ, de Klerk N, Burton P, Bower C (2012). Assisted reproductive technology and major birth defects in Western Australia. Obstet Gynecol.

[B56] Basso O, Baird DD (2003). Infertility and preterm delivery, birthweight, and caesarean section: A study within the Danish national birth cohort. Hum Reprod.

[B57] Hayashi M, Nakai A, Satoh S, Matsuda Y (2012). Adverse obstetric and perinatal outcomes of singleton pregnancies may be related to maternal factors associated with infertility rather than the type of assisted reproductive technology procedure used. Fertil Steril.

[B58] Marino JL, Moore VM, Willson KJ, Rumbold A, Whitrow MJ, Giles LC, et al (2014). Perinatal outcomes by mode of assisted conception and sub-fertility in an Australian data linkage cohort. PLoS One.

[B59] Kapiteijn K, de Bruijn CS, de Boer E, de Craen AJ, Burger CW, van Leeuwen FE, et al

[B60] Zhu JL, Obel C, Hammer Bech B, Olsen J, Basso O (2007). Infertility, infertility treatment, and fetal growth restriction. Obstet Gynecol.

[B61] Zhu JL, Basso O, Obel C, Bille C, Olsen J (2006). Infertility, infertility treatment, and congenital malformations: Danish ational birth cohort. BMJ.

[B62] Johnson MR, Riddle AF, Grudzinskas JG, Sharma V, Collins WP, Nicolaides KH (1993). Reduced circulating placental protein concentrations during the first trimester are associated with preterm labour and low birth weight. Hum Reprod.

[B63] Pinborg A, Loft A, Rasmussen S, Schmidt L, Langhoff-Roos J, Greisen G, et al (2004). Neonatal outcome in a Danish national cohort of IVF/ICSI and 10,362 non-IVF/ICSI twins born between 1995 and 2000. Hum Reprod.

